# Standardization of Pre-operative Evaluation to Improve the Outcome of Arteriovenous Fistula for Vascular Access of Hemodialysis: A Review of 700 Cases

**DOI:** 10.7759/cureus.45999

**Published:** 2023-09-26

**Authors:** Mukesh Suryawanshi, Palak Dutta, Rohit Ganduboina, Vikas Rajput, Shubhadarshini G Pawar

**Affiliations:** 1 General Surgery, Annasaheb Chudaman Patil Memorial Medical College, Dhule, IND; 2 General Medicine, Kyiv Medical University, Kyiv, UKR; 3 Medicine and Surgery, NRI Institute of Medical Sciences, Visakhapatnam, IND; 4 Nephrology, Shree Vighnaharta Superspeciality Hospital, Dhule, IND; 5 Medicine and Surgery, Bharati Vidyapeeth Medical College and Hospital, Pune, IND

**Keywords:** renal replacement therapy, primary failure, estimated glomerular filtration rate (egfr), end-stage renal disease, arteriovenous fistula

## Abstract

Background

With the advent of COVID-19, mortality rates of end-stage kidney disease (ESKD) rose by 37% which makes its treatment an important part of healthcare. Arteriovenous fistula (AVF) is linked to higher patient survival rates. Cimino-Brescia fistula is the most effective vascular access technique, but it has a high rate of primary failure (PF) and a several-week maturation period before it can be used. The current study aims to verify the preoperative evaluation in improving survival among AVF patients.

Methodology

The current study is a retrospective analysis of the hospital database from Jan 2022 to July 2023, with patients of a mean age of 60.2 years. The sample size was around 700, including the patients indicated for long-term hemodialysis (HD) with an estimated GFR of less than 20 mm/min/1.73m². Following AVF surgery, post-operative outcomes, PF, and maturation time were considered.

Results

Among the 757 AVF procedures, 588 (82%) were new cases, and 112 (16%) had prior AVF history on the same side. PF was observed in 126 (18%) AVFs, while 574 (84%) achieved maturation. Age at surgery did not correlate with PF. Male sex and brachiocephalic AVF (BCAVF) had lower PF rates, while female gender, non-BCAVF, and vascular chronic kidney disease (CKD) were independent predictors. Proximal fistulas had a higher failure risk (32%). During surgery, the PF occurred six times more frequently in patients with veins and arteries under 2 mm and without a bruit.

Conclusion

AVF maturation aims to achieve a functional AVF for easy dialysis, requiring meticulous vein selection, doppler vascular mapping, and a standardized process to reduce PF rates. Factors determining PF include thrill and bruit, flow rates, and comorbidities. These findings can help clinicians make informed decisions and improve outcomes for patients undergoing fistula surgery.

## Introduction

With the advent of COVID-19, end-stage kidney disease (ESKD) dialysis patients increased by 37% compared with the same time period in previous years [1]. The use of arteriovenous fistulas (AVFs) or grafts instead of central venous catheters (CVCs) increases ESKD survival and decreases infections, hospitalizations, and costs [2,3].

Following its description by Sribner in 1960, subcutaneous AVF for hemodialysis (HD) underwent multiple modifications, among which the Cimino-Brescia fistula between the radial artery and adjacent vein in 1966 was regarded as the best vascular access method [4]. The main disadvantage of AVF is its high primary failure (PF) rate and the delay in accessibility for use. A maturation period of many weeks is required to enable safe cannulation without jeopardizing the access's survival [5].

This present study aims to estimate PF rates, maturation times of common AVF, and long-term AVF survival to inform strategic access planning decisions; the present study is formulated as a single-center, single-surgeon observational study comparing preoperative vein and artery sizes, the presence of bruit and thrill on the operating table, flow rates, the location of the fistula, and related comorbidities for the post-operative outcome.

## Materials and methods

Study design

The current study is a retrospective analysis of patient databases from one high-volume dialysis facility between January 2022 and July 2023 at Shree Vighnaharta Superspeciality Hospital, Dhule, India, where around 1,700-1,800 dialysis sessions are performed annually. Patient databases were compiled for the study once the local Institutional Ethics Committee granted clearance (74IEC/ACPMMC/Dhule). Prior to the surgery, consent was obtained from patients. The sample size is estimated to be 700.

AVF surgery was performed on patients indicated for long-term HD by a vascular surgeon. The set inclusion criteria were age >18, ESKD with estimated glomerular filtration rate less than 20 mm/min/1.73m2, and patients posted for dialysis unit deemed long-term HD by a nephrologist [6]. The exclusion criteria were patients who didn't meet the inclusion criteria and those who denied consent. Several factors, including patient demographics, HD parameters, sonographic findings, and procedure details, were maintained in electronic databases with data on 700 patients.

Following a referral from a nephrologist, an ultrasonographer evaluated the patient's vascular system to see if they met the additional criteria for surgery. On clinical evaluation, the following basic venous criteria for surgery were used: distensible superficial cephalic/basilic vein, emptying test positive, non-tourtous course for at least 10 cm, and no history of recent intravenous cannulation. On color Doppler evaluation, there was no thrombosis or stenosis, no signs of surrounding inflammation, and no evidence of proximal blockage. The surgeon performed clinical testing on the vascular system in the forearms of both the dominant and non-dominant hands. A tourniquet test conducted at the mid-arm level aids in determining the patency of veins in both hands by looking at the dilated and non-tortuous section of the vein and venous valves.

The forearm vascular system was examined using grayscale and color Doppler ultrasound, showing vessel diameters, any luminal constriction, stenosis, obstruction, vessel wall thickness, plaques, calcifications, and vessel dissection. With the aid of color Doppler, arterial system flow mechanics, including flow direction, peak systolic and diastolic velocities, and flow characteristics like monophasic, biphasic, or triphasic, are largely examined. Following the preoperative assessment, the surgical procedure was performed at the proper anatomical site, side, and kind of anastomosis based on the information obtained (end-to-side or side-to-side).

AVF technique

At the time of clinical evaluation, arterial pulsation was assessed in the following ways: radial-nondominant, brachial-nondominant, radial-dominant, and brachial-dominant. The site with a good volume pulse was marked. The tourniquet was then placed around the mid-forearm of the non-dominant side to examine the cephalic vein. The tourniquet was then applied to the non-dominant side's midarm. If necessary, the same operation was repeated on the dominant side.

A well-dilated, non-tortuous vein segment roughly 10 cm long and passing the emptying test was sought. Valves were sometimes visible and labeled. Following that, a color Doppler evaluation was performed primarily to evaluate the arterial system. Vessel diameter, vessel wall characteristics such as plaque or calcifications, luminal narrowing or stenosis, luminal obstruction, and vessel dissection were all graded on a grayscale.

Color Doppler flow observations revealed peak systolic velocity and end-diastolic velocity, indicating the direction of blood flow and properties such as monophasic, biphasic, or triphasic flows. Additionally, if any obstructions were nearby (such as a thrombus), this was noted by an increase in turbulence. After completion, the location of side-to-side or end-to-side anastomosis was determined based on flow properties. If valves were near the anastomosis, the location was shifted by at least 5 cm.

Patients were operated on under regional anesthesia. Preoperative planning provided an idea of the skin incision, which varied from case to case. Following the skin incision, the pre-op strategy identified the vein and artery. While performing distal anastomosis, i.e., cephalic to the radial artery, if the artery ran beneath the tendon of the brachioradialis, as was the case if the anastomosis was a few cm proximal to the wrist crease, then the tendon was severed longitudinally. After completion, the artery was visible. If this was not done, the vein had to go around the tendon when the artery returned to its normal position causing a kink and significantly reduced flow.

If a superficial to deep proximal forearm perforator was identified on Doppler evaluation in proximal AVF, the same perforator was ligated with non-absorbable suture material. Ligation of the perforator was critical; otherwise, it would form a short loop with low resistance after anastomosis. Furthermore, it would cause two issues: first, it would prevent superficial veins from arterialization, and second, it would cause persistent limb edema.

The anastomosis was completed continuously with polypropylene. Absorbable sutures were used to close the skin. This gave us four potential sites for AVF creation. Compared to synthetic grafts, AVF created with this technique was far superior.

Post-operative care

Post-surgery, the AV fistula was monitored for patency and maturity to rule out early or late problems. First, a physical examination was done by palpation to check for the thrill in the fistula; later, an auscultatory approach was used to detect the bruit along the veins to verify the fistula's patency and rule out any stenosis or dilations.

Maturation time was defined by both clinical and duplex ultrasound methods, clinically maturation time is defined by time from the date of procedure to the date of the sixth consecutive dialysis with two needles; this criterion is especially true for patients with CVC [7], another definition is via the rule of 6s' describing at least 600 ml/min blood flow rate, 6 mm diameter cannulation zone, <6mm depth, and 6 cm length of cannulation zone [6].

Predetermined criteria define PF as the inability to perform dialysis using two needles for six consecutive sessions [7].

Statistical analysis

After collection, data were presented in a Microsoft Excel sheet (Microsoft Corporation, Washington, United States); data were managed using variable tests on STATA version 15 (StataCorp LLC, Texas, United States). PF was analyzed using descriptive analysis and univariate analysis, and logistic regression was used to analyze PF and maturation rate to determine risks of procedure failure, with a p-value of <0.05 being considered significant. All means were described with standard deviations.

## Results

Study population

During the period between January 2022 and July 2023, 757 AVF operations were performed; 700 of these were gathered for this study.

The study's data was split into variables in Microsoft Excel, statistical analysis was performed, and the findings were presented. Table 1 displays population characteristics by AVF type. In 392 cases, the radiocephalic vein was utilized, the brachiocephalic vein in 217, and the brachiobasilic vein in 91.

**Table 1:** Population characteristics by type of AVF AVF: arteriovenous fistula, RCAVF: radiocephalic arteriovenous fistula, BBAVF: brachiobasilic arteriovenous fistula, BCAVF: brachiocephalic arteriovenous fistula, CKD: chronic kidney disease

Variable	RCAVF	BCAVF	BBAVF	p-value
Male	270 (68.88%)	98 (45.16%)	37 (40.65%)	<0.001
Type 2 diabetes	166 (42.34%)	84 (39%)	30 (33%)	0.52
Vascular CKD	47 (12%)	30 (14%)	8 (8.79%)	0.38
Auto-immune CKD	43 (10.96%)	31 (14.28%)	17 (18.68%)	0.63
Polycystic CKD	20 (5.1%)	7 (3.22%)	4 (4.39%)	0.02
Post-renal CKD	32 (8.16%)	15 (6.91%)	3 (3.29%)	0.31
Pre-dialysis	220 (56.12%)	87 (40.09%)	9 (9.89%)	<0.001
Previous AVF	20 (5.10%)	56 (25.80%)	40 (43.56%)	<0.001
Access surgeon	349 (89.03%)	177 (81.56%)	77 (84.61%)	0.05

PF and maturation time

Table 2 summarizes the results of descriptive and univariate analyses performed to determine PF rates among different patient categories. The PF occurred in 126 (18%) of the AVF, while 574 (84%) matured, and the maturation time had an interquartile range of 6-14.6 weeks. Age at surgery was unrelated to PF: the mean age for AVF with PF was 58.74. The patient's state prior to dialysis and the performing surgeons were not associated with PF. Brachiocephalic AVF (BCAVF) and men had considerably lower PF. Patients with vascular CKD had higher PF. The PF rate was slightly greater in pre-existing AVFs (p=0.89). Female gender, non-BCAVF, and vascular CKD were found to be independent predictors of PF in multivariate analysis (Table 2). Average blood urea and serum creatinine levels were substantially lower in individuals with successful fistulas than in patients with PF (<0.001) (Table 3).

**Table 2:** Descriptive analysis and univariate analysis of PF AVF: arteriovenous fistula, RCAVF: radiocephalic arteriovenous fistula, BBAVF: brachiobasilic arteriovenous fistula, BCAVF: brachiocephalic arteriovenous fistula, CKD: chronic kidney disease, PF: primary failure

Variable	Mature (n=574)	PF (n=126)	Total	p-value
Male	348 (85.92%)	57 (14.07%)	405 (57.85%)	<0.001
Female	226 (76.61%)	69 (23.38%)	295 (42.14%)	
Type 2 diabetes	201 (71.78%)	79 (28.21%)	280 (40%)	0.42
Non-diabetic	315 (75%)	105 (25%)	420 (60%)	
Age ≤65	210 (75%)	70 (25%)	280 (40%)	0.79
Age 65-80	221 (71.75%)	87 (28.24%)	308 (44%)	
Age >80	79 (70.53%)	33 (29.46%)	112 (16%)	
Vascular CKD	58 (68.23%)	27 (31.76%)	85 (12.14%)	<0.001
Non-vascular CKD	468 (76.09%)	147 (23.90%)	615 (87.57%)	
Polycystic CKD	26 (83.87%)	5 (16.12%)	31 (4.42%)	0.36
Non-polycystic kidney	522 (78.02%)	147 (21.97%)	669 (95.57%)	
Autoimmune CKD	72 (79.12%)	19 (20.87%)	91 (13%)	0.86
Non-autoimmune	481 (78.98%)	128 (21.01%)	609 (87%)	
Post-renal CKD	36 (72%)	14 (28%)	50 (7.14%)	0.27
Non-post-renal CKD	481 (74%)	169 (26%)	650 (92.85%)	
RCAVF	290 (73.97%)	102 (26.02%)	392 (56%)	<0.001
BCAVF	185 (85.25%)	32 (14.74%)	217 (31%)	
BBAVF	70 (76.92%)	21 (23.07%)	91 (13%)	
On dialysis at AVF op	291 (79.94%)	73 (20.05%)	364 (52%)	0.60
Pre-dialysis at AVF op	249 (74.10%)	87 (25.89%)	336 (48%)	
Previous AVF same side	85 (75.89%)	27 (24.10%)	112 (16%)	0.89
No AVF same side	452 (76.87%)	136 (23.12%)	588 (82%)	

**Table 3:** Blood urea and serum creatinine value

Test	Mean	SD
Blood urea level (mg/dl)		
Successful fistulas	85.38	40.42
Failed fistulas	118.65	32.84
Serum creatinine level (mg/dl)		
Successful fistulas	5.95	2.49
Failed fistulas	8.9	2.21

Using logistic regression (Table 4), it emerged that the risk of failure was higher in the distal fistula (32%) than in the proximal fistula (12%) (p-value <0.001).

**Table 4:** Logistic regression of PF risk

Variables	Odds ratio	95% CI	p-value
Site of fistula			
Proximal	0.4706	0.45-2.21	<0.001
Distal	0.3158	0.34-2.85	0.41
Artery <2mm			
Yes	1		
No	0.47	0.47-2.09	0.06
Vein <2 mm			
Yes	1		
No	6.021	0.51-1.95	<0.001
Bruit			
Yes	1		
No	6.228	0.46-2.12	<0.001
Thrill			
Yes	1		
No	5.671	0.54-1.84	<0.001

PF was seen six times more among patients with veins <2 mm than among patients with veins >2 mm (p-value <0.001). PF was seen more among patients with arteries <2 mm than patients with arteries >2 mm (p-value 0.06) (Table 4).

PF is 6.2 times more in patients with no bruit heard on the operative table than in patients with bruit (p-value <0.001). PF is 5.6 times more in patients with no thrill palpated on the operative table than in patients with thrill (p-value <0.001) (Table 4).

## Discussion

Because of its safety, long-term effectiveness, and fewer issues than other techniques such as CVC and AVG, the National Kidney Foundation recommends AVF as the preferred access for long-term HD in patients with chronic or end-stage renal disease. As a result, creating a native AVF on the arm or forearm is a superior option to prosthetic grafts and CVC [5,8,9]. However, when HD is required urgently, and no other vascular access is available or has failed, CVC is the preferred method of temporary access [10].

This is a prospective study of 700 patients with ESKD who are on dialysis. The participants in our study ranged in age from 48 to 73. Patients with various kinds of CKD were included in the study. Wilmink et al. and Sahasrabudhe et al. previously conducted studies that found PF was higher in vascular CKD cases and females than males, yielding results similar to ours as mentioned in Table 2 [7,11].

The most general operating procedure was the construction of the distal radio-cephalic fistula, first described by Brescia et al. in 1966 [4]. This approach is still the gold standard for vascular access for HD [12], accounting for 392 (56%) of our surgical procedures. BCAVF (31%) and BBAVF (13%) were also produced. Our study's PF was comparatively lower at 18% on average, compared to earlier studies with PFs of 23% and 21.2%, respectively [7,11]. The reasons for increased PF in women are unclear; differences in vessel diameters do not explain them [13]. AVF generated after patients began dialysis had much greater survival than those created before. The pre-dialysis state during AVF operation yielded a PF of 25.89%, whereas the patient who was on dialysis during AVF operation yielded a PF of 20.05%. As a result, the findings of our study and the Wilmink et al. study [7] contradict each other, claiming that the pre-dialysis state had no effect on PF.

One of the most difficult obstacles in AVF is the several weeks required for maturity [7]. The median maturation time in our study was IQR 6-14.6 weeks, which was better than the median maturation time in Wilmink et al.'s study, which was IQR 7.4-16.3 weeks [7].

According to the American Institute of Ultrasound in Medicine Practice Guideline 2011, arterial diameters of less than 2 mm and venous diameters of 2.5 mm are associated with a significant failure rate. This study also shows that less than 2 mm venous widths are related to significantly higher failure rates (p-value <0.001). However, in our study, an arterial diameter of 2 mm was not found to be significant [14,15].

The risk of failure was lower in proximal fistulas (12%) in our series, which was statistically significant (p-value 0.001). This finding is supported by a study by Sultan et al. [16], which found that four years of primary efficient patency was better with proximal fistulas than distal fistulas.

Serum creatinine and blood urea may also have little impact on fistula success, as levels in patients with successful fistulas were considerably lower than in patients with PF, as verified by a study by Sahasrabudhe et al. [11]. Our research revealed a strong connection between PF and bruit heard at the end of surgery or the thrill felt on the operating table following dressing. When the bruit and thrill were absent, PF increased by 6.2 and 5.6 times, respectively.

While our study, conducted within a single center and involving a solitary surgeon, offers valuable insights, it is imperative to recognize the potential limitations inherent to this specific setting. In contrast, one study [17] found that trainees and consultants achieved comparable outcomes when case selection, decision criteria, and operating methods were standardized. Our study shares common ground with this prior research, reinforcing the notion that standardized approaches can yield consistent results, even in different settings.

The study's limitations were no reliable data on the reasons for AVF failure or the number of interventions required to achieve AVF maturation. PF caused by early thrombosis is indistinguishable from failure to mature. According to a big Scottish study [18], most PF is assumed to be caused by a failure to mature. There is still room for improvement demonstrated by the low number of pre-dialysis treatments on non-maturing AVF and the high success rate. The fastest maturation rate observed in BCAVF as seen in Table 2 could be attributed to vessel size. However, there is no data to support this [7].

## Conclusions

The ultimate goal of AVF maturation is to achieve a functional AVF that enables easy dialysis. This outcome depends on several factors, including meticulous vein selection, precise vascular mapping using Doppler, and following a standardized process to reduce the PF rate. Failure to achieve a functional anastomosis can lead to an unsatisfied nephrology colleague and a frustrated patient. Other factors determining PF include the presence of thrill and bruit, flow rates, and comorbidities like diabetes, CKD, and blood urea/serum creatinine levels. A vein diameter of 2 mm or less has a high failure rate and requires a proximal location or alternative access placement. The presence of bruit and thrill during surgery is a crucial indicator of successful fistula surgery, but this finding has yet to be documented in the literature. These findings can help clinicians make informed decisions and improve outcomes for patients undergoing fistula surgery.
